# Epistemic inclusion in the Qanuilirpitaa? Nunavik Inuit health survey: developing an Inuit model and determinants of health and well-being

**DOI:** 10.17269/s41997-022-00719-4

**Published:** 2022-12-22

**Authors:** Christopher Fletcher, Mylène Riva, Marie-Claude Lyonnais, Annie Baron, Ida Saunders, Melody Lynch, Marie Baron

**Affiliations:** 1 Department of Social and Preventive Medicine, Université Laval, Quebec City, QC Canada; 2 Centre de recherche du CHU de Québec – Université Laval, Quebec City, QC Canada; 3 Canada Research Chair in Housing, Community and Health, Institute for Health and Social Policy, Montreal, QC Canada; 4 Department of Geography, McGill University, Montreal, QC Canada; 5 Nunavik Regional Board of Health and Social Services, Kuujjuaq, QC Canada; 6 Kuujjuaq, Canada; 7 Université Laval, Quebec City, QC Canada

**Keywords:** Epistemology, Inuit, Concepts, Social determinants of health, Culture, Qualitative research, Épistémologie, Inuits, concepts, déterminants sociaux de la santé, culture, recherche qualitative

## Abstract

**Objective:**

At the request of Nunavik Inuit health authorities and organizations, the Qanuilirpitaa? 2017 Nunavik regional health survey included an innovative “community component” alongside youth and adult epidemiological cohort studies. The community component objective was to identify and describe community and culturally relevant concepts and processes that lead to health and well-being.

**Methods:**

A qualitative, community-based research process involving workshops and semi-structured interviews was used to generate a corpus of data on health concepts and processes specific to Inuit communities in Nunavik. Thematic analysis and repeated community validation allowed for the identification of three key dimensions of health salient to Inuit experience and eight community-level health determinants.

**Results:**

The health model consists of three linked concepts: *ilusirsusiarniq*, *qanuinngisiarniq*, and *inuuqatigiitsianiq*, which reflect distinct dimensions of Inuit health phenomenology. The determinants *community*, *family*, *identity*, *food*, *land*, *knowledge*, *economy*, and *services* were generated through analysis and reflect community-level sources of health and well-being.

**Conclusion:**

The development of the culturally grounded health models and determinants is an exercise of epistemic inclusivity through which researchers and Indigenous communities may form new and equitable paths of knowledge creation.

## Résumé

**Objectif:**

À la demande des instances et des organisations de santé inuit du Nunavik, une « composante communautaire » novatrice a été intégrée à l’enquête régionale de santé Qanuilirpitaa? 2017 en parallèle aux études épidémiologiques menées auprès des jeunes et des adultes. L’objectif de la composante communautaire était d’identifier et de décrire les concepts et les processus, pertinents d’un point de vue communautaire et culturel, qui contribuent à la santé et au bien-être.

**Méthodes:**

Un processus de recherche adoptant une approche qualitative et axée sur la communauté a été employé, en menant notamment des ateliers et des entrevues semi-structurées, pour créer un corpus de données sur la conceptualisation et les processus de santé propres aux communautés inuit du Nunavik. L’analyse thématique des données et la validation des résultats par les communautés ont permis d’identifier trois dimensions clés de la santé qui sont essentielles à l’expérience qui en est faite par les Inuit, ainsi que huit déterminants de la santé au niveau communautaire.

**Résultats:**

Le modèle de santé est composé de trois concepts étant intrinsèquement liés, *ilusirsusiarniq*, *qanuinngisiarniq* et *inuuqatigiitsianiq*, et qui structurent les dimensions les plus importantes de la phénoménologie de la santé inuite. Les déterminants suivants sont ressortis de l’analyse des données et reflètent les sources de santé et de bien-être au niveau communautaire : *la communauté, la famille, l’identité, la nourriture, le territoire, le savoir, l’économie* et *les services*.

**Conclusion:**

L’élaboration de modèles et de déterminants de la santé fondés sur la culture est un exercice d’inclusion épistémique qui permet aux chercheurs et aux communautés autochtones de former de nouvelles voies vers une création plus équitable des connaissances.

## Introduction

Health research is increasingly attentive to Indigenous culture, ontology, philosophy, historical experience, and lived reality—a conceptual and epistemological developments evident in, among many other innovations, the cultural adaptation of research tools to better “fit” the populations of concern, a growing body of work on cultural safety and security in health service provision, and the identification of health determinants that are relevant to and evident in Indigenous communities. This intellectual change has occurred alongside more than two decades of investment in the development of the careers of Indigenous health researchers by national and provincial granting bodies, universities, and research centres (Reading, 2003). The net effect is a growing openness towards the knowledge systems of Indigenous peoples, recognition that expertise is inherent to Indigenous communities, and a blurring of the sociological lines between the researchers and those studied, ultimately fostering the decolonization of the conceptual and methodological processes of knowledge creation.

Cultural models of health have been articulated by and with Indigenous peoples in Canada generally, and for specific nations, communities, and subpopulations within them (Parlee & O’Neil, 2007; Ward et al., 2020; Delormier et al., 2017; Radu et al., 2014; Allen et al., 2020; Ward et al., 2021, Healey, 2017; Cueva et al., 2021; Martin, 2012). The exploration of Indigenous models of health in public health applications finds its origin in the work of Mason Durie, Maori psychiatrist, academic and health advocate. Durie’s influential model of Maori health has been adapted and adopted across health services in *Aotearoa* (New Zealand), and its example has since inspired many other projects in Pacific nations and Indigenous societies around the world (Durie, 1985, 1999, 2004). As an example of the Indigenization of public health in a developed, wealthy, “Western” country with a significant Indigenous population, we see in it the potential for knowledge creation in locally meaningful and culturally contributive ways. Health interventions that are adapted, derived, and otherwise shaped by Indigenous ways of being are widely thought to hold considerable promise to be effective, safe, and empowering. In Canada, the great diversity of Indigenous peoples, nations, and communities has fostered many original exercises of cultural acknowledgement, inclusion, and reconciliation with paradigms of health research, program development, service provision, and administrative control.

This paper describes the development of a holistic model of Inuit health and well-being and the identification of eight community-level health determinants undertaken as part of the Qanuilirpitaa? 2017 Nunavik Health Survey (Qanuilirpitaa hereafter)—a large-scale regional health survey undertaken in the Inuit territory of Quebec, Canada. We situate this work within an emerging critical discourse around the place of Indigenous epistemology, knowledge, and expertise within the Indigenous and broader global health fields (Prussing, 2018; Bhakuni & Abimbola, 2021). The foundational work of Spivak (1988) on “epistemic violence” to describe processes through which people living under colonial regimes come to be known through selective and discriminatory knowledge practices that contribute to their displacement from traditional lands and cultural ways of being is particularly salient. In this light, the development and application of culturally and linguistically derived Indigenous health models is a means to repatriate the existential grounds of health to Indigenous homelands and lifeways. In a reflection of Inuit preference for fostering positive and constructive discourses around health and well-being, we use the concept of *epistemic inclusion* to signal a step towards cross-cultural intellectual innovation, social justice, Indigenous capacity, and equity in health research. It is not by accident that the project we describe here was called for by Inuit once the chance to authorize a new health survey was taken up in Nunavik. Even if the survey itself exists within and serves a provincial-level health system and administration, the potential for shaping the way health is imagined, understood, and ultimately lived and fostered is enhanced by the development of models that are part of Inuit experience.

Indigenous cultural experts often have little reason to engage with institutional processes and publishing venues when their audiences of concern are community members, nation governments, and the broader Indigenous intellectual worlds. Indeed, it has often been argued that the formal Western academic and governmental research worlds are to be avoided if cultural integrity and control is to be maintained. Decolonization of such environments is a prerequisite to effective inclusion of Indigenous peoples, worlds, and knowledge systems (Tuck & Yang, 2012). Inuit self-determination in research is an important part of the Inuit Tapiriit Kanatami (ITK; the national organization representing Inuit in the four land claims regions of Northern Canada) position on research conduct that calls for substantial changes in control over research decision-making and resource allocation and control (ITK, 2018). The movement to intellectual inclusion and diversity in academic environments is an important and challenging epistemological change that is occurring in universities and research centres that face countervailing—and often incommensurate—pressures to increase academic productivity measured in research monies received, numbers of publications produced, and their placement in prestigious journals. This paper is meant to contribute to bridging the intellectual worlds of the Inuit culture and community with the university and advocate for approaches that are grounded in listening between and among people.

### A mandate conferred

Attention to the history of public health research in the north was important in the conceptualization of the Qanuilirpitaa survey. Surveys of Inuit health have been undertaken for over 100 years (Fletcher, 2017), making them one of the most consistent causes of interaction between Inuit and southern researchers. In the 1940s, population health data began to be sought out to support the extension of government programs in the north that were seen to be essential in the context of steadily worsening health conditions of people in many northern regions. These efforts intensified in the 1950s when northern administrations responded to very difficult conditions faced by Inuit as a result of a number of intersecting conditions including declines in caribou populations, family ruptures resulting from the evacuations of people afflicted with tuberculosis to sanitoria in southern Canada, and a distressingly high rate of infant mortality. Many northern communities were in effect created through these early public health efforts signalling a very significant change in Inuit lives and livelihoods. Health survey data have thus been at the heart of major social transformations for Inuit which themselves incurred many other changes and impacts. We can conceptualize public health responses over time in the north (such as housing programs, well baby clinics, maternal health education, and enforced schooling) as physical, ontological, and philosophical wedges introduced between people and their ways of thinking, doing, and experiencing health. This is not to discount their efficacy or pertinence, but rather to point to the implication of public health in broader social processes of cultural alienation and colonization that have had profound impacts on population health and well-being. The intertwining of public health knowledge creation and practice in the north has had significant impacts for Inuit.

The James Bay and Northern Quebec Agreement signed in 1975 (Government of Quebec, 1976) included provisions for the development of health services under the control of people in the region including the creation of the Nunavik Regional Board of Health and Social Services (NRBHSS). While the NRBHSS is a public institution and part of the provincial health care system, given the overwhelming majority of Inuit in Nunavik, it is a de facto Inuit organization. Since the 1980s, the NRBHSS has provided direction and participated in population health surveys in the region. The earliest was the Plasannouq survey (Blanchet et al., 1992), which established a baseline of public health data on which the Nunavik Public Health was founded. This was followed by the Santé Québec 1994 (Jetté & Santé Québec, 1995) and Qanuippitaa? 2004 (INSPQ, 2008) surveys.

The Qanuilirpitaa survey is at once a continuity of these earlier surveys and a beginning of a new direction. The need to expand the scope of the survey data beyond the traditional epidemiological focus on mental and physical health and include an exploration of health as perceived and lived by Nunavimmiut was formulated at an International Inuit health survey workshop held in Kuujjuaq in 2012. Building on this consensus, in 2014, the NRBHSS Board of Governors made the decision to go forward with the Qanuilirpitaa survey that would include two traditional epidemiological cohort studies of youth (16–30 years old) and adults (over 30 years, many of whom were surveyed in 2004), and a “community component” that would take in this new perspective. The goal of the community component was to describe health from the perspective and experience of people in their home communities. From this broad description, a series of health determinants were to be produced. The community component was conceived to make research responsive and recognizable to community members and ultimately as a bridge to link research results to community mobilization for health.

The objectives of the community component were to:

- describe Inuit cultural concepts of health and well-being in relation to health determinants and community living;
- better understand how conditions and resources in communities contribute to the health of people;
- identify sources of strength and resilience in each community in response to health challenges;
- using the framework developed, measure and describe community health and well-being across all 14 communities in Nunavik; and
- provide data and information to develop community action plans and interventions to respond to the health and well-being needs of Nunavik.

The broad and exploratory nature of the mandate called for a range of methods to be used in an inductive process to describe the cultural and social contexts of health and well-being from a community perspective. Subsequently, a team of researchers with qualitative, quantitative, community-based, and action research method experience was formed under the guidance of the NRBHSS.

## Methods

The health survey was a very large project that saw the CCGS Amundsen icebreaker used as a platform for the research team to visit each of the 14 communities of Nunavik between August and October of 2017. The community component employed a participatory, community-based approach to describe health concepts and determinants. The research process took place over a period of 1.5 years beginning in June 2016, during the intensive data-gathering phase onboard the Amundsen in 2017 through to a final validation meeting in October 2018. An iterative and emergent research design process allowed for learning at each phase of the project to shape the specific methods and objectives used in the next. Knowledge creation during the project was thus cyclical and cumulative. Community and regional institutional validation of findings was undertaken at several intervals. The findings were also reviewed and commented upon by a Data Management Committee, which had ultimate authority over the diffusion of Qanuilirpitaa survey results.

The project was undertaken over five phases: (1) document preparation; (2) community concept mapping; (3) interviews; (4) analysis; and (5) validation. We briefly describe these below.

### Phase 1. Document preparation

Two threads of literature were explored during the initial phase. The first was a review of Inuit language dictionaries and lexicons, and related documents for health-related terminology. We developed lists of health-related terms and definitions, and used the terminology as a basis for initial discussions with language experts on the project team. From these, we created a short list of terms and concepts to be used in the second phase of the research. Indigenous language vitality is widely understood to be a positive indicator of health and the means through which health knowledge and conditions are best transmitted. Epistemic inclusion is thus first and foremost accomplished through working with the language world of the people concerned.

The second strand of documentation reviewed was Indigenous- and Inuit-specific health determinant frameworks and relevant reports from Nunavik. Reading and Wien’s (2009) report on Indigenous health determinants is a cornerstone document in the field and served to orient our development of community determinants. The Parnasimautik report (Makivik Corporation et al., 2014), a region-wide consultation with organizations and institutions in Nunavik, offers a comprehensive overview of issues in the region, many of which correspond to a health determinant framework. Finally, a report by Inuit Tapiriit Kanatami (2014) provides a comprehensive description of Inuit health determinants. We synthesized these reports into a working set of themes for exploration in subsequent phases.

### Phase 2. Community concept mapping

The second phase of research consisted of workshops in two communities in Nunavik. The communities were chosen to be inclusive of the perspectives of people on both the Hudson and Ungava coasts—geographic distinctions that have cultural and ecological relevance for Nunavimmiut. In both cases, community leadership had previously indicated their interest in participating in this phase of the project. Team members also had long-term working relationships with people in the chosen communities, which facilitated the integration of the research team and the comfort level of participants in the workshops.

The workshops took place over 1.5 days. Participants were suggested by community leaders and purposefully selected based on their knowledge and interest in the subject. A diversity of men, women, youth, and elders were present in both cases. A total of 10 people in the first community and 11 in the second participated. Participation was voluntary and participants were compensated for their time. A natural, conversation style of sharing was used throughout. Methodologically, it was important to have conversations in “everyday” community spaces that were not overtly medical nor marked practically or conceptually as Qallunaaq (non-Inuit) spaces. Thus, workshops were held in community buildings that were regularly used for diverse community activities and were thus familiar and comfortable spaces for participants.

The workshops were audio recorded, and flip charts and other visual aids were used during the meetings. A mix of Inuktitut and English was used throughout the meetings, and translation was provided to participants and team members as needed. Conversations were guided by three basic questions: (1) What is health/well-being and what does it mean to you? (2) What makes you healthy and well? and (3) What in your community makes you healthy?

The language terminology collected in phase 1 was used at various points during the workshops and helped to encourage conversation and clarify ideas. We noted that physical objects such as lists, pens, Post-it notes, and other items on the meeting table allowed quieter participants to take notes, draw, and remain engaged with the workshop. The physical space, props, and objects used in meetings like this one are rarely remarked on in the health research literature but are nonetheless methodologically significant. How people are placed, the social dynamics of speaking, and the physical comfort of sharing space with others are all phenomenological dimensions of discourse with significant influence on the quality and depth of conversation.

### Phase 3. Community interviews, August–October 2017

In August 2017, the CCGS Amundsen began its voyage at Kuujjuarapik in southern Hudson Bay, sailed north to Ivujivik and then south along the coast of the Hudson Strait and Ungava Bay with stops at all 14 communities of Nunavik along the way. While the ship anchored nearby, members of the research team were ferried from the ship to the community each morning and returned on board at the end of the day. Time available in each community varied between 1 and 4 days depending on the sailing schedule of the Amundsen and other factors. During the visits, two to three team members met with representatives of different community organizations, local initiatives and community associations, key community members, and others interested in Inuit health and well-being. The objective of the visits was to deepen the understanding of the sources and determinants of health specific to each community and to identify and map the community-level health resources. We used semi-structured interviews, questionnaires, and community asset mapping techniques. Given the short time available in many of the communities, a follow-up visit to eight of the 14 communities was undertaken in January 2018 so that all the people and organizations targeted in the process could be contacted. Ultimately, the research team were able to interview people and map services in all 14 communities.

#### Semi-structured interviews

Between three and seven key community members, suggested by community partners, were purposefully selected for interviews in each community. We interviewed a total of 64 people. A broad range of the population was sought to gather a variety of perspectives on the sources of health and well-being. Two researchers, with the support of a community member/interpreter as needed, undertook the interviews in the language of choice of the respondent. Interview topics, which were determined in concept mapping workshops with the communities, included community life, family relationships, relations with services offered, access to food, spirituality, and economy. The interviews were conducted through naturalistic exploration of themes relevant to the respondent’s work and roles in the community. Interviews lasted between 45 min and 2.5 h.

### Phase 4. Analysis

Recordings from the community workshops and in-depth interviews were transcribed verbatim in English, and thematic analysis was undertaken to identify relevant concepts and to organize the data. We coded transcripts using NVivo 12 qualitative analysis software package (QSR International Pty Ltd., 2018). From these, a preliminary version of community health themes and an Inuit model of health were drawn up. The workshop data and interview material gathered in the 14 communities were also transcribed, coded, and thematically analyzed using NVivo 12. Two team members coded individually and compared codes to increase rigour. A preliminary thematic analysis produced 12 discrete categories relevant to community health.

### Phase 5. Validation

In October 2018, community validation sessions were held in the two communities where the initial workshops took place. The initial 12 themes were presented and discussed by a group of participants, many of whom were involved in the original workshops. Linguistic terminology was refined, and the meanings of the terms and the overall structure of the model were explored in more detail. The 12 themes were presented and discussed during a regional meeting of the mayors of Nunavik in Kuujjuaq in May 2018. Staff of the NRBHSS were also consulted on the themes. A reanalysis of the themes based on the feedback from these meetings produced a final set of eight health determinants relevant to people in the communities of Nunavik.

## Results

### The IQI model of health

**Fig. 1:**
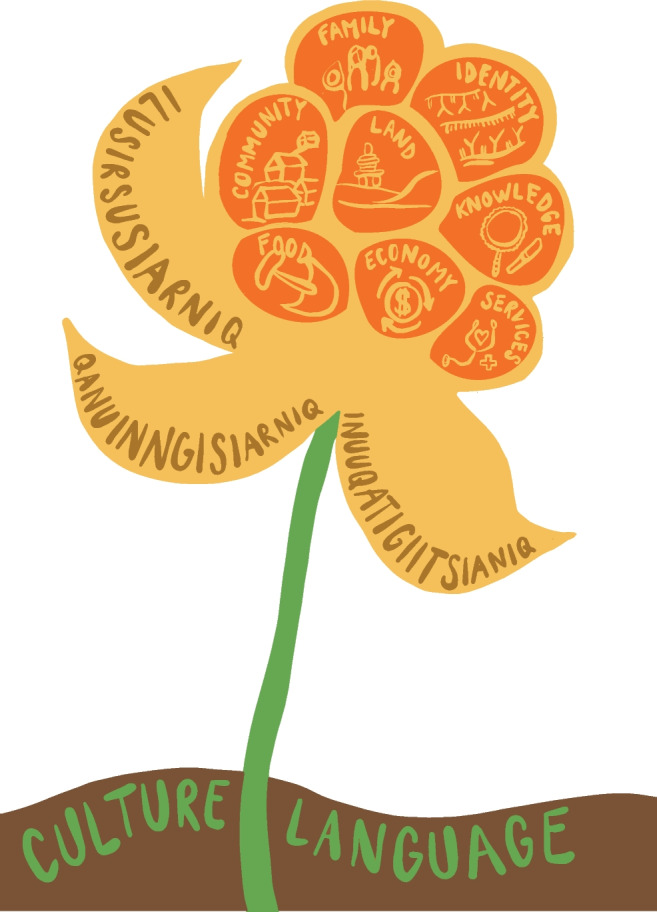
The IQI model

The Inuit model of health and community-level determinants is presented in graphic form in Fig. 1. The foundation of health is found in Inuit *uqausiq* (language) and *iluqiutiq/piusiq* (culture). These, as we heard from people in the community workshops, are the basis for life for Inuit affording the knowledge and skills required to survive and thrive in the north, and the social grounding of relations between people, in families and beyond. To live well is to communicate and work effectively together. Through these, people are raised from infancy to become autonomous and capable individuals who are sure of themselves and their abilities. Piusiq references the specific ways that community life unfolds, the “core of things” as one workshop participant put it.

Out of the foundation of language and culture, three key concepts emerge to form the basis of the model of Inuit health: *ilusirsusiarniq*, *qanuinngisiarniq*, and *inuuqatigiitsianiq*. The roots of these terms are widely used in Inuktitut, and appear in documents and reports from other regions of Inuit Nunangat (Healey & Tagak Sr, 2014; Laugrand et al., 2001). The term “IQI model” has spontaneously and rapidly been taken up by people working across the health system in Nunavik, and we follow this format hereafter. The brief synthesis of the meanings of each term presented below glosses over the nuance of the language.

*Ilusirsusiarniq* is roughly equivalent to “bodily health” in English and was invoked in discussions about cancer, air quality, nutrition, and other physical ailments. The semantics in Inuktitut convey a condition of normal and unremarkable capacity, of being without pain, infirmity, or sickness and being able to carry out the tasks required of everyday life. The concept also carries the sense that health and the growth and ageing of the body follow a natural and intended progression across the lifespan, “things taking their intended form” as one respondent put it (see also Therrien, 1995, for a similar discussion of *Ilusiq*, the root of *Ilusirsusiarniq*).

*Qanuinngisiarniq* is a broadly defined sense of well-being that encompasses feelings of being unworried, without pain, comfortable, free of emotional distress, and happy. The concept evokes a sense of psychological and spiritual peace of mind, calmness, fulfilment, and being able to move forward and carry on with ease. *Qanuinngisiarniq* is manifested in peoples’ lives when they can achieve their goals, live comfortably in their homes, share joy and affection with their families and loved ones, and be free of excessive grief, mourning, and worry. To experience *qanuinngisiarniq* is to not be weighed down by worries, to face others with openness, and to be able to recognize and share happiness. It was described in interviews as a feeling of lightness and freedom.

While proximal, *qanuinngisiarniq* and *ilusirsusiarniq* should not be taken to be analogous to mental and physical health in Western medical nosology and culture. *Qanuinngisiarniq* and *ilusirsusiarniq* denote culturally salient phenomenological states of being and not merely the presence or absence of disease.

The final term in the model, *inuuqatigiitsianiq*, is an ideal form of social well-being that is manifest through relatedness, caring, and productive working relations which rely on the quality of interpersonal relations among *nunaliit*—people who share the same place. Good relationships with spouses and partners, family members, friends, neighbours, and people in the community are essential to create the conditions of health and well-being. Poor relations can trigger and accentuate tensions between people, fomenting anger, frustration, and (in extreme forms) violence. Within families, considerable attention is paid to fostering *inuuqatigiitsianiq* in relationships over time. Families that are inclusive of all members, are not afflicted by addictions, do not face excessive financial or other worries, and have overcome prior difficulties have the ground for healthful living. At the community level, good leadership that is reassuring and effective brings the sense of quality of shared living to the fore. *Inuuqatigiitsianiq* is inclusive of relations with non-Inuit and people with whom Inuit interact in the south, for schooling, work, and government.

Supportive family and community members nurture the sense of *qanuinngisiarniq* by reducing worries, solving problems, supporting others through difficult times, and encouraging productive action. *Ilusirsusiarniq* is also fostered through the social support and mutual aid that allows food to be shared, work to be done, travel to be safe, children to be well cared for, and hunting to be successful. Ultimately, the three terms should be seen as mutually entwined and reinforcing. Each of the three concepts overlaps with the others to produce a conception of health and well-being that is dynamic and multidimensional. Epistemically, they call attention to the contexts in which good actions, positive sentiment, and appreciation of family and community members take place. Collectively, they point to ways of being healthy that are specific and recognizable to Inuit. To begin to frame a discussion of health and the results of health surveys within these opens an avenue to epistemic inclusion in health research, practice, and services.

### The eight community determinants of health

The final step in the analysis was the identification and description of eight determinants of health: *community*, *family*, *identity*, *food*, *land*, *knowledge*, *economy*, and *services*, which are briefly described below. Each determinant and synthesis draws on the thematic analysis of the workshops and interview data and has been validated by community members and regional health authorities.

*Community* encompasses the social, physical, and built spaces of the villages of Nunavik. This determinant is composed of the dynamic social web of individuals, family, genders, ages and origins of the people, and their ways of being together in groups within the physical setting of each municipality. Community is social and physical and includes the infrastructure of everyday community life, including municipal services, housing, public spaces, institutions, roads, and transportation networks that people use in their daily lives.

*Family* is the key organizing principle of community life and includes the kinship ties and affective relations between family members across generations. The importance and concern for the lives of young people was very commonly raised in the interviews. People also stressed the Inuit way of recognizing and being with relatives in the extended family. Inuit cultural customs, like the practice of customary adoption, the naming of babies after loved ones and the recently deceased, and other practices of inclusiveness, connecting, and parenting, were said to shape the “sense of family” that people rely on and in which people may find great joy. Societal changes associated with living in contemporary villages have undermined some traditional roles and responsibilities.

*Identity* as a subject of popular discourse is a relatively recent phenomenon associated with inclusion of Inuit into the national body politic. As non-Inuit have moved to the north and north-south mobility has increased for Inuit, a questioning of the nature of what it means to be Inuit has become common. An insecurity of identity is understood to create the conditions for psychological and ontological insecurity that manifests in tragedy and early death. Conversely, learning Inuit norms, culture, and skills serves to anchor people in a historical continuum of culture, territorial occupation, and practices in the villages of Nunavik today. As a shared set of ideas, identity serves as a marker of inclusion and belonging expressed through language, knowledge, history, childhood and youth experiences, and interactions with public and private agencies and institutions, distinct from southern Canadian or Qallunaaq culture.

*Food* is the physical manifestation of Inuit knowledge and skills. The production, sharing, and consumption of country foods is seen as a particularity to Inuit and a source of great pride and pleasure in all aspects. Food is a physical necessity of life that carries equally important social, cultural, and economic dimensions. The production of food is fundamentally linked to the land of Nunavik and the ice and waters of the sea to which all communities are connected. Food as a health determinant is inextricably linked to knowledge of the land and fauna. Store-bought foods are also key for health and the costs of southern groceries and equipment important.

*Land* is the place on which people live and thrive. The experience of health, healing, and well-being in Nunavik is closely linked to the land in material and ideological terms. The land is home to the animals, plants, and resources that have always sustained Inuit. Precise knowledge of the landscape and its resources is required to make them available to people, and a broad range of skills are required to survive when facing harsh weather. While Nunavimmiut no longer rely solely on the land for all the necessities of life, much is still gathered from the land and waters of the region. The land retains its significance as the cultural foundation of the people.

*Knowledge* is the precursor to effective action supporting health. Specific knowledge changes over the life of an individual and is modulated by gender, family, community, and individual experiences. Today, there are many sources of knowledge. Among them are family and community sharing, schools, vocational training, television, and internet. Effective use of knowledge is a quality of good leadership and governance. It is also a prerequisite to any efforts to move and hunt on the land.

*Economy* is broadly defined to include the different sources and means of monetary income and goods, including the natural resources. Economy is thus the intersection of community development, jobs, and land-based resources, as well as the transformation and (re)distribution of resources within families, communities, and beyond. Economy acts on the three dimensions of the IQI model in many ways.

*Services* feature strongly in the discussions of community-level sources of health and well-being. These include the full range of health and social services in the communities, at the two regional hospitals in Nunavik, and in southern Quebec. Services also encompass the informal sources of healing, counselling, and assurance that are found in other people and organizations, including churches and community activities like sewing centres and youth activities.

## Discussion

Efforts to produce a cultural model of health are by design and necessity reductionist and partial. Nevertheless, exploring and recognizing the specificity of Indigenous understandings of health is an important pathway to equitable and decolonized care. Cultural models can influence the organization of health care systems and service delivery in ways that are responsive to and reflect the values and experiences of the populations they serve (Allen et al., 2020). The IQI model builds on this approach by describing in detail a relatively small number of key concepts and articulating their intersections with each other and with community-defined determinants of health. The IQI model thus simplifies and structures cultural complexity with implications for cross-cultural work in complex health systems. Historically, northern health care delivery has been inseparable from colonial processes and attendant social transformation, yet largely unconscious of its own role in these processes. Inuit concepts and models support reflexive and equitable practice for those who are new to the north, and a familiar ground for Inuit for whom such ideas are simply normal parts of everyday life. This duality is particularly important when roles of people in health care delivery are stratified by ethnicity as is the case in Inuit Nunangat where there are very few Inuit health care professionals. Attention to cultural models and community-defined determinants recognizes the unique historical trajectory of Inuit as they have been confronted by broad-scale colonial and neo-colonial processes, widely recognized as the most encompassing of determinants of Indigenous health today (Czyzewski, 2011; Paradies, 2016).

This paper draws from a tradition of research on the semantics of health-related terminology in Inuit communities (Therrien, 1987, 1995; Therrien et al., 2001; Kirmayer et al., 1997, 2008). It builds on these efforts by linking the research outcomes to community health mobilization efforts by Inuit-led health authorities. More broadly, our research contributes to large-scale efforts to situate health care within Inuit social and structural contexts. For example, Healey (2017) describes a number of concepts germane to Inuit in Nunavut and concludes that, were the health care system to animate the value of *Piliriqatigiinniq*, or working for the common good, it would be better able to recognize and incorporate the diverse contributions of community members and professionals to health. Likewise, following Tagalik’s (2015) observation that for Inuit, health is inherent to the collective and cultural process of making a human being, the model recognizes health as inseparable from ontology.

Identifying key determinants of Indigenous peoples’ health contributes to understanding the historical origins and structural persistence. The eight community determinants identified here are a unique contribution to the literature on Indigenous health determinants. It builds on the foundation work of Reading and Wien (2009) who were the first to propose a systematic and multilevel overview of health determinants for Indigenous peoples in Canada. A set of 11 Inuit-specific health determinants were subsequently developed in 2014 by Inuit Tapiriit Kanatami. Both Reading and Wien and ITK drew from academic literature and expert knowledge to produce key social determinants. As can be seen in Table 1, the eight determinants of the IQI model share elements of the ITK and Reading and Wien models. An important difference is in the methodology and process of validation in our work. The determinants identified in the IQI model were derived from close work in the 14 communities of Nunavik, thus privileging local experience and knowledge in the framework. It has also been through a rigorous community validation process that ensures the accuracy and pertinence of the model and determinants, thus lending credibility to the structure. The contribution this study makes is thus conceptual and methodological. A limitation in both the ITK and our study is the reliance on English translation and dissemination. In an attempt to mitigate this shortcoming in the IQI model, we have worked closely with Inuit language experts to identify the appropriate terminology, explore the semantics of the terms, and preserve the essence of the meaning in the analysis and presentation of the model.

**Table 1:** Social determinants of health

Reading and Wien (2009)	ITK 2014	Qanuilirpitaa
Health behaviours	Quality of early childhood development	Community
Physical environments	Culture and language	Family
Employment and income	Livelihoods	Identity
Education	Income distribution	Food
Food insecurity	Housing	Land
Health care systems	Personal safety and security	Knowledge
Educational systems	Education	Economy
Community infrastructure, resources, and capacities	Food security	Services
Environmental stewardship	Availability of health services	
Cultural continuity	Mental wellness	
Colonialism	Environment	
Racism and social exclusion		
Self-determination		

Finally, an important innovation in this study is situating the eight determinants in a matrix with the three foundational conceptual elements of the IQI model grounded in a cultural phenomenology of health and well-being. Each determinant can be explored and understood within the three dimensions of the IQI model, thus increasing the nuance and precision for understanding the myriad factors that influence health in the community. Working with community-level determinants within the structure of the IQI model recognizes Inuit ways of knowing, thinking, and experiencing health and opens the door to culturally coherent and safe approaches to analyzing and acting on health at the community level.

## Conclusion

The community component of the Qanuilirpitaa? Nunavik health survey foregrounds the knowledge, ideas, and ways of experiencing health proper to people and communities. The IQI model is meant to convey and represent a culturally familiar set of sentiments, meanings, and embodied conditions that constitute health in a way that contributes to innovative and effective actions for health.

The focus of the model is on social interaction, capacity, and comfort; moving with efficacy, fluidity, and grace; and being with others in comfort, joy, and care. The IQI model is thus a phenomenology of living well, rather than a register of disease and the cumulative impact of inequity. The IQI model presents a shift from a taken-for-granted position about what constitutes health towards one that is grounded in Inuit epistemology, ontology, language, and lived experience. This is part of an ongoing and necessary process of repositioning health surveillance, epidemiology, and skills in ways that contribute to the potential of people and communities. Such process (re)imagines and constructs new socio-political and resource paradigms. These changes are required in a post-colonial disease environment where both suffering and wellness are fully social, historical, and biological. Ultimately, the IQI model and determinants of community health emerged through methodology of multifaceted and attentive listening that was made possible by a combination of Inuit empowerment to direct research and an openness to other ways of being well.

## Contributions to knowledge

What does this study add to existing knowledge?

- A new model of health grounded in Inuit language and epistemology underpins the identification of a set of community-level health determinants. Methodologically, it makes contributions to how to undertake cross-cultural research that is shaped by Indigenous epistemology and lived reality.

What are the key implications for public health interventions, practice, or policy?

- The IQI model provides a conceptual framework on which to build effective and locally meaningful health programs, interventions, and policy.

## Data Availability

NA

## Code availability

NA
